# Magnetic‐Field‐Enabled Ultrafast Quench Synthesis of Single‐Atom Catalysts for Efficient Anion Exchange Membrane Water Electrolysis

**DOI:** 10.1002/anie.2977516

**Published:** 2026-07-08

**Authors:** Shenghua Chen, Yaqiong Su, Kaixin Liang, Yiqing Sun, Fangkun Fan, Yifei Shen, Lingyou Zeng, Kai Xi, Chunhui Xiao, Xiaobo Zheng, Dingsheng Wang, Guoxiu Wang, Ya‐Ling He

**Affiliations:** ^1^ School of Chemistry, National Innovation Platform (Center) For Industry‐Education Integration of Energy Storage Technology, School of Chemical Engineering and Technology Xi'an Jiaotong University Xi'an P. R. China; ^2^ Shanxi Xinhua Chemical Equipment Research Institute Co., Ltd. Taiyuan P. R. China; ^3^ Center For Clean Energy Technology, School of Mathematical and Physical Sciences, Faculty of Science University of Technology Sydney Sydney Australia; ^4^ Department of Chemistry Tsinghua University Beijing P. R. China

**Keywords:** ultrafast synthesis, single‐atom catalysts, magnetic‐field‐enabled quench, anion exchange membrane water electrolysis

## Abstract

Single‐atom catalysts (SACs) play a critical role in diverse catalytic applications, but their efficient synthesis remains a significant challenge. Herein, we develop an ultrafast magnetic‐field‐enabled quench (MFEQ) strategy to synthesize a series of M_1_/G‐FeO_x_ (M═Ni, Fe, Co, Ir, Ru, and Pt) SACs within a few seconds. Using Ni_1_/G‐FeO_x_ as a proof of concept, this method leverages the rapid quenching of thermally incandescent Fe foam into an Ni‐containing ethanol solution, triggering simultaneous graphene formation and Ni anchoring. The Ni_1_/G‐FeO_x_ catalyst shows exceptional alkaline oxygen evolution reaction (OER) performance, operating at 200 mV for 10 mA cm^−2^ and sustaining 105 mA cm^−2^ for 330 h without degradation. Notably, the Ni_1_/G‐FeO_x_‐catalyzed anion exchange membrane water electrolysis (AEMWE) device exhibits a low voltage of 1.86 V at 1.0 A cm^−2^ and 600 h long‐term stability. Density functional theory (DFT) calculations and experiments reveal that the strong electronic interactions between Ni_1_/G and FeO_x_ contribute to the optimized electronic structure and reduced energy barrier. Techno‐economic analysis (TEA) highlights the superior energy efficiency of the MFEQ method, which requires only US$19.2 in energy expenditure to synthesize 1 kg of SACs. This work provides new insights into the ultrafast fabrication of SACs.

## Graphical abstract

We report a universal magnetic‐field‐enabled quench strategy for the ultrafast synthesis of diverse single‐atom catalysts within seconds. This simple and low‐cost method yields highly active catalysts with excellent performance in anion exchange membrane water electrolysis.

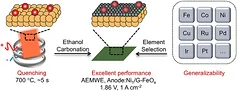

## 1 Introduction

Improving the atomic economy of participating in chemical reactions and ensuring maximum utilization of rare catalytic materials are core goals of sustainable chemistry [1, 2]. Heterogeneous single‐atom catalysts (SACs), which combine atomically dispersed metal centers with a customizable coordination environment, demonstrate the potential to achieve both goals in energy catalysis [3, 4, 5, 6]. The catalytic performance of SACs is intimately governed by the local coordination environment of the active sites, which, in turn, is critically determined by the synthetic route [7, 8]. Consequently, the practical deployment of SACs is inherently linked to their preparation methodologies, underscoring the importance of developing simple and general synthetic strategies to facilitate their real‐world application [9].

Currently, researchers have developed a variety of synthesis methods for SACs, including atomic layer deposition [10, 11], high‐temperature pyrolysis [12, 13], wet‐chemical methods [14, 15, 16], and coordination assembly strategies [17, 18]. However, these synthesis routes often involve complex fabrication procedures, including the preparation of precursors and multiple post‐treatment steps, or require specialized and expensive equipment. More importantly, for most SACs, particularly carbon‐supported SACs, their synthesis heavily relies on prolonged high‐temperature calcination to achieve the carbonization process, which is both time‐consuming and energy‐intensive [19]. Therefore, developing a low‐cost, facile, and ultrafast synthesis strategy for SACs is of paramount importance for advancing both fundamental research and industrial applications.

Here, we report an ultrafast and highly efficient magnetic‐field‐enabled quench (MFEQ) strategy for the rapid construction of SACs. This method enables the successful synthesis of a series of M_1_/G‐FeO_x_ (M═Ni, Fe, Co, Ir, Ru, and Pt) SACs within only a few seconds, highlighting its exceptional scalability and generality. Taking Ni_1_/G‐FeO_x_ as a representative example, Fe foam serves as both the structural scaffold and the Fe source. Under magnetic‐field induction heating, the surface Fe atoms are rapidly oxidized to form FeO_x_, creating abundant anchoring sites. Immediately afterwards, the incandescent Fe foam is quenched in a Ni‐containing ethanol solution, where the ethanol is instantaneously carbonized into graphene. The freshly formed graphene simultaneously captures and stabilizes the Ni ions, ultimately yielding atomically dispersed Ni sites on the graphene‐coated FeO_x_ framework. The obtained Ni_1_/G‐FeO_x_ catalyst demonstrates superior alkaline oxygen evolution reaction (OER) activity with a low overpotential of 200 mV at 10 mA cm^−2^ and ultra‐long stability up to 330 h without obvious decay at the current density of 105 mA cm^−2^. Moreover, the assembled anion exchange membrane water electrolysis (AEMWE) device using Ni_1_/G‐FeO_x_ as an anode exhibits high activity with a low cell voltage (1.86 V @1000 mA cm^−2^) and excellent stability for 600 h. This study introduces an ultrafast, economically attractive route for generating SACs suitable for AEMWE and reveals new conceptual insights into the rapid, scalable production of advanced energy‐functional materials.

## 2 Results and Discussion

**FIGURE 1:**
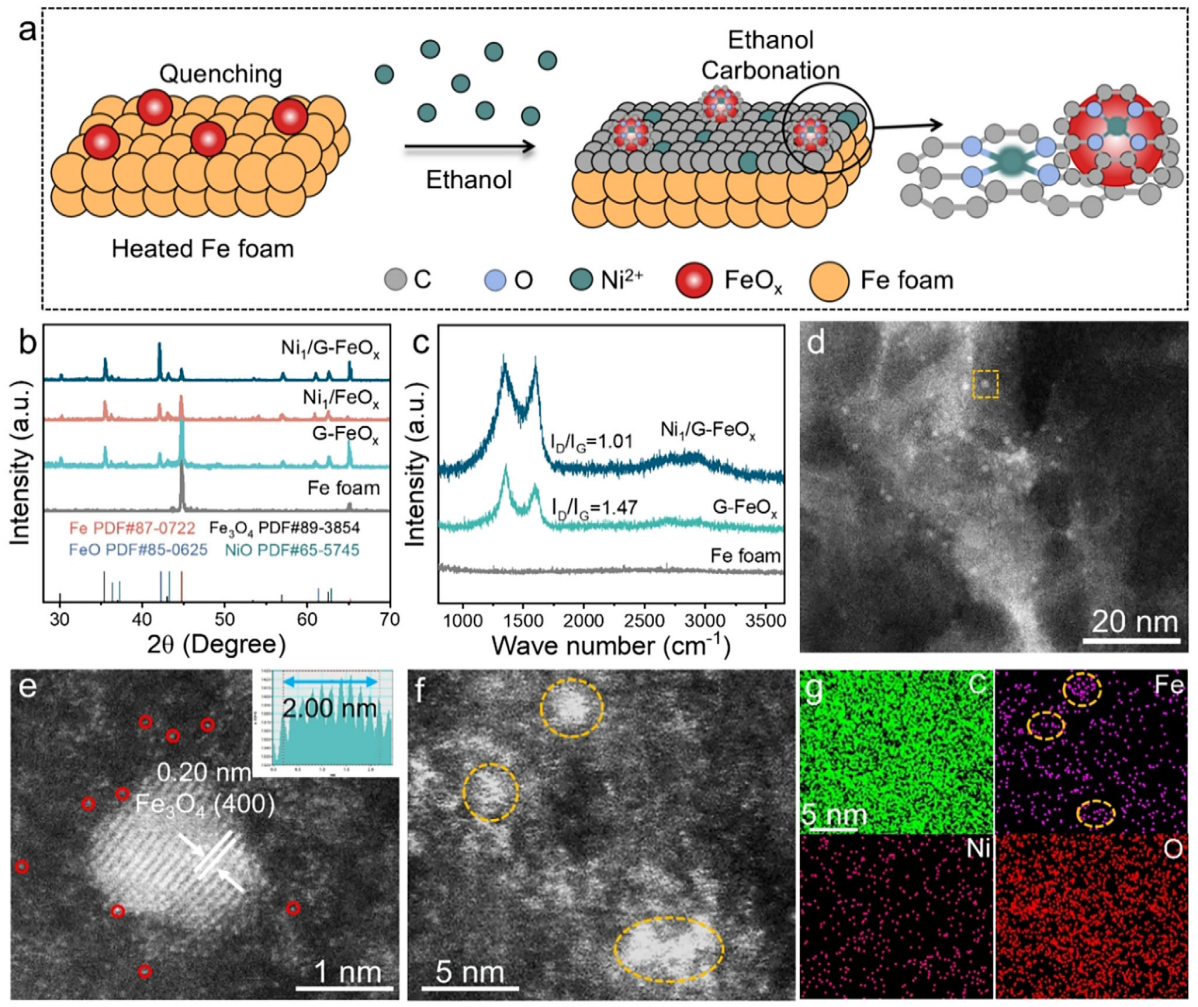
Synthesis process and structural characterizations of the Ni_1_/G‐FeO_x_ catalyst. (a) Schematic illustration of the MFEQ synthesis process for Ni_1_/G‐FeO_x_. (b) XRD patterns of Fe foam, G‐FeO_x_, Ni_1_/FeO_x_, and Ni_1_/G‐FeO_x_ together with the standard PDF reference patterns of Fe_3_O_4_, FeO, and NiO. (c) Raman spectra of Fe foam, G‐FeO_x_, and Ni_1_/G‐FeO_x_. (d, e) HAADF‐STEM images of Ni_1_/G‐FeO_x_. (f, g) STEM‐EDS elemental mapping images of Ni_1_/G‐FeO_x_.

The overall MFEQ synthetic protocol is illustrated in Figures 1a and S1. In a typical procedure, the cleaned Fe foam (Figure S2) was rapidly heated in air to thermal incandescence via inductive heating (∼5 s). During this process, thermally driven surface oxidation formed a thin layer of iron oxide. The incandescent Fe foam was then immediately quenched in an ethanol solution containing nickel nitrate at room temperature. Fundamentally, the successful stabilization of isolated Ni single atoms (Ni_1_/G‐FeO_x_) is governed by a synergistic mechanism that combines thermodynamic anchoring and rapid kinetic freezing [20, 21, 22, 23]. Upon immersion, the transient high temperature triggers incomplete carbonization of ethanol, forming a defective graphene that retains oxygen‑containing functional groups (Figure S3). These oxygen‑containing functional groups serve as efficient thermodynamic anchoring sites for Ni ions, forming stable Ni‐O interfacial configurations that immobilize Ni as isolated single atoms. Concurrently, the rapid liquid quenching instantly deprives the system of the thermal driving force required for long‐range surface diffusion, thereby kinetically freezing the atomic dispersion and precluding Ostwald ripening (Figures 1b–d, S4 and S5) [24]. This method involves ultrafast heating and cooling processes completed within approximately 5 s, making it highly attractive for large‐scale, low‐cost production.

The X‐ray diffraction (XRD) pattern confirms the surface oxidation of the Fe foam during the magnetic‐field‐enabled heating process, as evidenced by the appearance of Fe_3_O_4_ and FeO diffraction peaks (marked as FeO_x_, Figure 1b). Notably, no characteristic peaks corresponding to Ni or NiO are observed, indicating the absence of crystalline Ni‐ or NiO‐related phases. Raman spectra further reveal two shoulder peaks corresponding to the D band (1342 cm^−1^) and G band (1582 cm^−1^), which are associated with graphitic carbon containing edge‐plane defects and defect‐free sp^2^ carbon domains, respectively (Figure 1c) [25, 26]. These features confirm the formation of graphene on the Fe foam surface after ethanol quenching. Upon the introduction of the Ni source, the *I*_D_/*I*_G_ ratio decreases from 1.47 to 1.01, suggesting an decrease in carbon defect density. Such defect‐rich carbon provides abundant anchoring sites for stabilizing isolated Ni atoms.

High‐angle annular dark‐field scanning transmission electron microscopy (HAADF‐STEM) images (Figures 1d,e and S6) reveal the presence of abundant FeO_x_ nanoparticles with an average size of ∼3 nm uniformly dispersed on the graphene surface. A measured lattice spacing of 0.20 nm corresponds to the (400) plane of Fe_3_O_4_ (Figures 1e and S7). Notably, numerous isolated bright spots are observed surrounding the Fe_3_O_4_ nanoparticles, which can be assigned to atomically dispersed Ni species formed during the rapid quenching process. Energy dispersive spectroscopy (EDS) spectra show that the mass loading of Ni atoms could reach ∼6 wt.% (Figure S4). Moreover, some nanoclusters are observed on the support, which can be attributed to the formed FeO_x_ species through the EDS elemental mapping analysis (Figures 1f,g and S8). To further verify the microscale distribution of metal species, local electron energy loss spectroscopy (EELS) was performed (Figure S9a,b). The characteristic Fe and O signals were markedly stronger than those of Ni, suggesting that Ni is atomically dispersed with an extremely low local loading. Additionally, high‐resolution STEM‐EDS line‐scan analysis was conducted on a selected region containing isolated bright spots but no observable FeO_x_ nanoparticles (Figure S9c,d). The selected region mainly contains C, O, and Ni signals, whereas the Fe signal remained nearly invisible. Notably, the localized Ni signal precisely coincided with the positions of the isolated bright spots in the STEM image. Additionally, the generation of graphene can be further confirmed by the characteristic (002) plane of graphitic C with a d‐spacing of ∼0.36 nm (Figure S10) [27]. Thus, combining magnetic‐field‐enabled heating with ethanol quenching enables the successful synthesis of the Ni_1_/G‐FeO_x_ catalyst within a few seconds.

**FIGURE 2:**
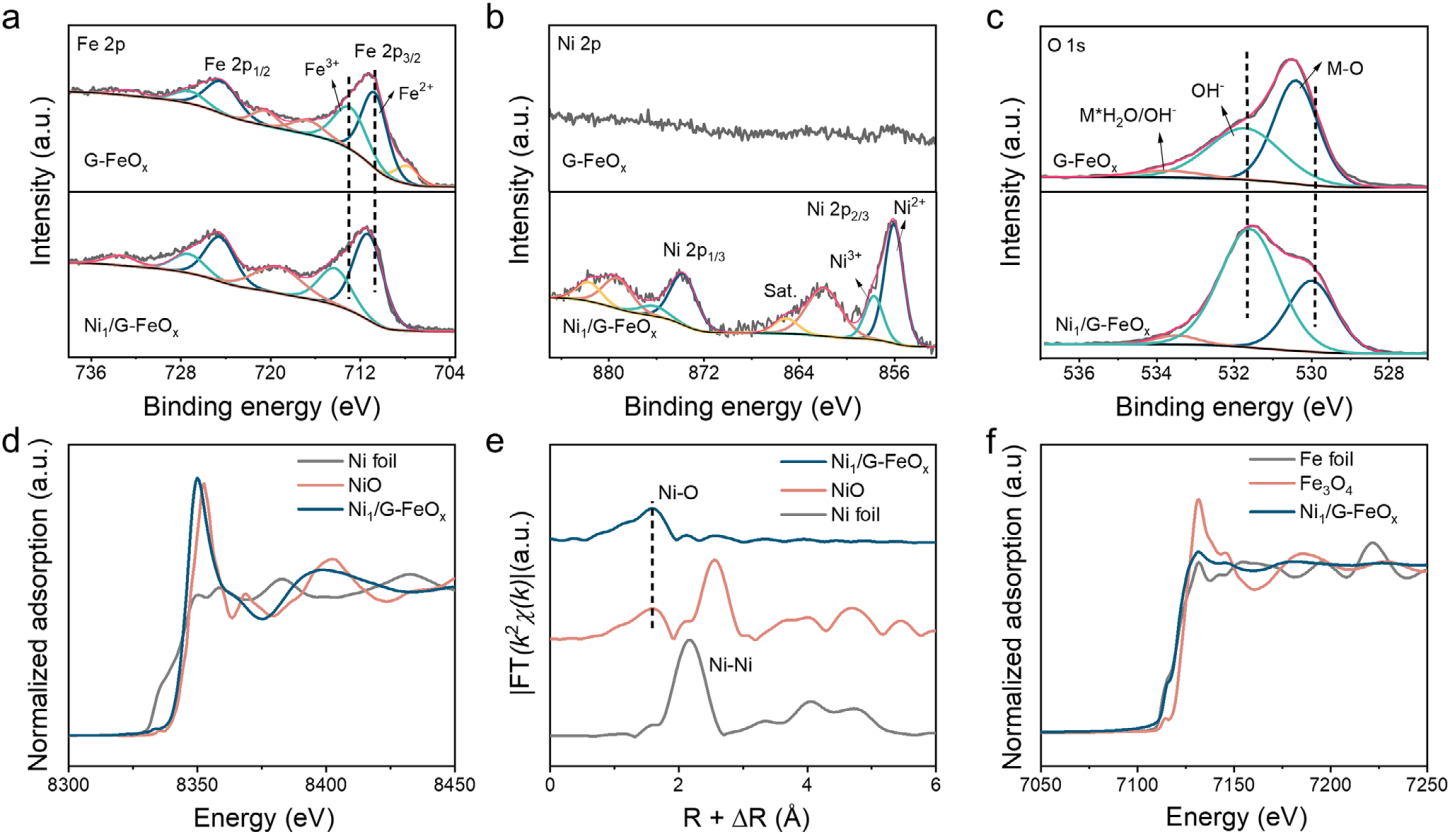
Electronic structure characterizations. High‐resolution (a) Fe 2p, (b) Ni 2p, and (c) O 1s XPS spectra of G‐FeO_x_ and Ni_1_/G‐FeO_x_. (d) Normalized Ni K‐edge XANES spectra, (e) FT‐EXAFS spectra of Ni_1_/G‐FeO_x_, NiO, and Ni foil. (f) Normalized Fe edge XANES spectra.

X‐ray photoelectron spectroscopy (XPS) was conducted to reveal the surface electronic structure of Ni_1_/G‐FeO_x_ (Figure S11). The high‐resolution Fe 2p XPS spectra show that the Fe^2+^ and Fe^3+^ species in Ni_1_/G‐FeO_x_ exhibit higher binding energies than those in pristine G‐FeO_x_ (Figure 2a). As displayed in Figure 2b, the G‐FeO_x_ sample lacks any detectable Ni signal, as expected. Meanwhile, the O 1s spectra reveal a decreased binding energy for the lattice oxygen peak of Ni_1_/G‐FeO_x_, indicating enhanced electron density around lattice O upon single Ni atom incorporation. Together with the positive shift in the Fe 2p spectra, these findings suggest that introducing Ni_1_/G drives charge redistribution within the Fe‐O framework, thereby causing electron depletion at Fe sites and electron accumulation at coordinated oxygen atoms (Figure 2c). These indicate a strong synergistic electronic interaction between Ni_1_/G and the FeO_x_, leading to significant local charge redistribution within the Ni_1_/G‐FeO_x_ system.

To further elucidate the electronic structure of Ni_1_/G‐FeO_x_, X‐ray absorption near‐edge structure (XANES) and extended X‐ray absorption fine structure (EXAFS) analyses were performed. As shown in Figure 2d, the Ni K‐edge XANES spectrum of Ni_1_/G‐FeO_x_ displays an absorption edge position similar to that of NiO, while being markedly shifted to higher energy relative to metallic Ni foil. The results indicate that the Ni species in Ni_1_/G‐FeO_x_ are in an oxidized state, with an average oxidation state close to +2. The corresponding EXAFS spectrum (Figure 2e) reveals a dominant Ni‐O coordination peak at approximately 1.6 Å (without phase correction), while no distinct Ni‐Ni metallic coordination peak is observed near 2.2 Å. These findings provide strong evidence for the absence of metallic Ni clusters or nanoparticles in Ni_1_/G‐FeO_x_. Wavelet‐transform EXAFS (WT‐EXAFS) analysis of Ni_1_/G‐FeO_x_ shows intensity peaks solely attributable to the Ni‐O scattering path, further confirming the atomic‐level dispersion of the Ni species (Figure S12). Quantitative EXAFS fitting was subsequently performed to determine the local coordination environment of the Ni atoms (Figure S13 and Table S1). The fitting results reveal a Ni‐O coordination number of 3.74 around the Ni center, consistent with a tetrahedral coordination configuration, together with an average Ni‐O bond length of approximately 2 Å. Therefore, these conclusions demonstrate the Ni‐O_4_‐type single‐atom coordination structures.

As shown in Figure 2f, the Fe K‐edge XANES spectrum of Ni_1_/G‐FeO_x_ exhibits an absorption edge that is slightly higher in energy than that of Fe foil, yet significantly lower than that of Fe_3_O_4_. Although this observation may appear counterintuitive, it arises from the bulk‐sensitive nature of X‐ray absorption spectroscopy (XAS), which measures the averaged oxidation state of both the surface FeO_x_ species and the metallic Fe foam in Ni_1_/G‐FeO_x_. To verify this interpretation, we carried out linear combination fitting of the Fe K‐edge XANES spectrum for Ni_1_/G‐FeO_x_. The fitting results quantitatively support our hypothesis, revealing that Fe foil contributes the dominant fraction (66.2%), whereas FeO_x_ accounts for 33.8% (Figure S14). The Fe K‐edge EXAFS spectrum of Ni_1_/G‐FeO_x_ displays a pronounced peak at ∼1.53 Å, corresponding to the first‐shell Fe‐O coordination (Figures S15 and S16). Importantly, compared with the Fe_3_O_4_ reference, the Fe‐O coordination environment in Ni_1_/G‐FeO_x_ becomes broader and less ordered, indicating the presence of partially disordered or defective FeO_x_ domains induced by the ultrafast quenching process and graphene coupling. Furthermore, the fitting results indicate that the Fe‐O coordination number in Ni_1_/G‐FeO_x_ is approximately 1.3, with an average bond length of approximately 2.04 Å (Table S2). Additionally, a peak located at ∼2.7 Å is observed, which is attributable to the second‐shell Fe‐O‐Fe scattering [28]. A small shoulder near ∼2.2 Å, resembling the Fe‐Fe coordination feature in Fe foil, further corroborates the XANES fitting results. Similar conclusions can be drawn from the wavelet transform (WT) EXAFS contour maps of the Fe K‐edge (Figure S17).

**FIGURE 3:**
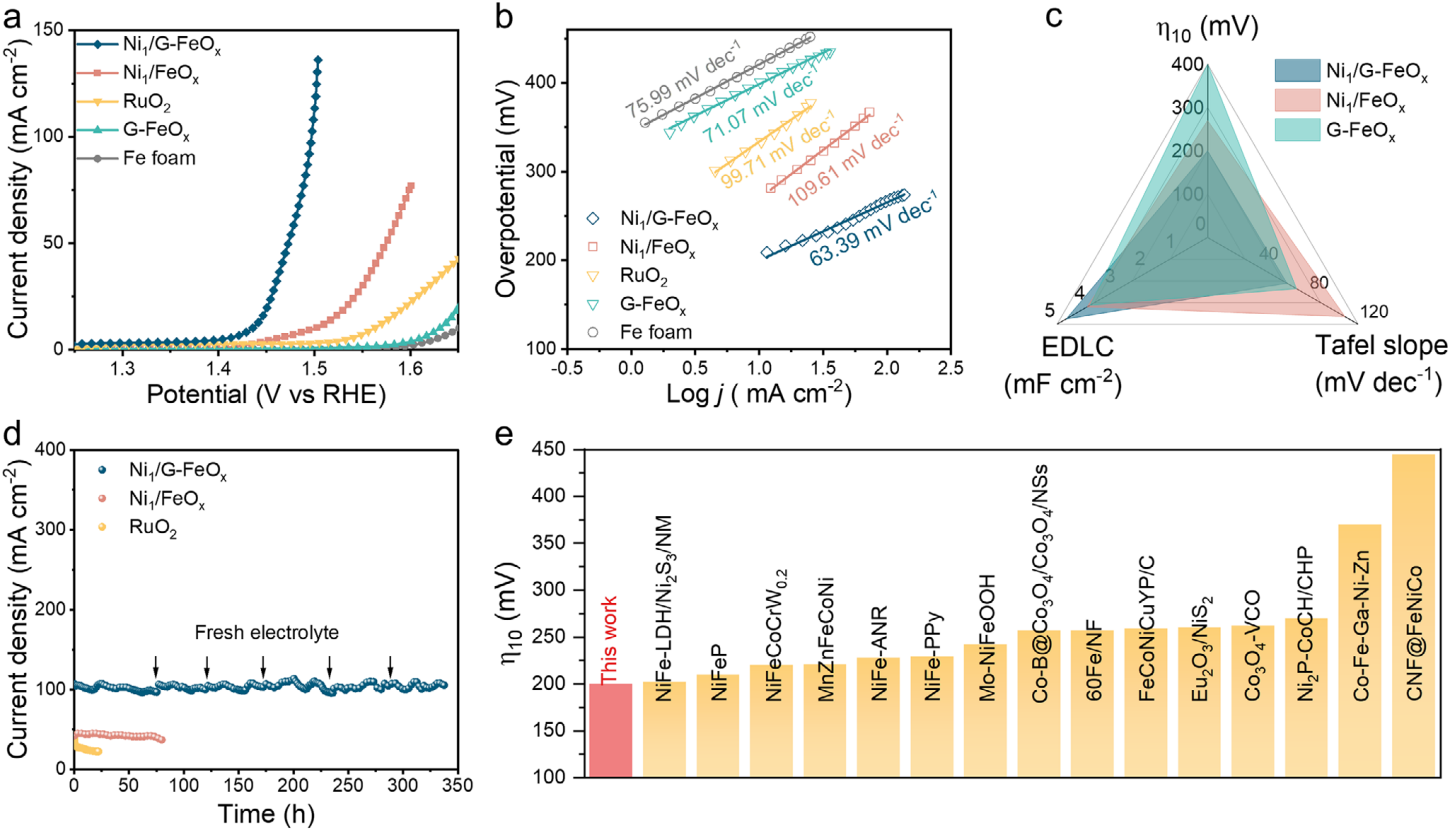
OER activity of the Ni_1_/G‐FeO_x_ catalyst. (a) LSV curves of Ni_1_/G‐FeO_x_, Ni_1_/FeO_x_, G‐FeO_x_, and the reference samples. (b) Tafel curves. (c) The radar charts of catalytic activities for Ni_1_/G‐FeO_x_, Ni_1_/FeO_x_, and G‐FeO_x_. (d) Stability tests of the Ni_1_/G‐FeO_x_, Ni_1_/FeO_x_, and RuO_2_. (e) Comparison of OER performance with other recently reported electrocatalysts.

The OER performance was tested in 1.0 M KOH using a standard three‐electrode system (Figure S18). First, various parameters, including quenched time and salt solution, were considered to optimize the OER activity (Figures S19–S22). The linear sweep voltammetry (LSV) curves show that the Ni_1_/G‐FeO_x_ catalyst requires only a small overpotential (*η*) of 200 mV to reach a current density of 10 mA cm^−2^, outperforming that of G‐FeO_x_, Ni_1_/FeO_x_ catalyst (Figure 3a). Notably, the Ni‐free G‐FeO_x_ catalyst exhibits much lower OER activity relative to Ni_1_/G‐FeO_x_, which may suggest these surface‐bound single‐Ni sites are the dominant active centers. Interestingly, no sign of a transport‐limited regime appeared in the studied potential range, reaching over 120 mA cm^−2^ at *η* = 271.5 mV. We then compared electrochemical kinetics via the Tafel analysis (Figure 3b). The smallest Tafel slope of 63.39 mV dec^−1^ is observed for the Ni_1_/G‐FeO_x_ catalyst, which is lower than that of G‐FeO_x_ (71.07 mV dec^−1^), Fe foam (75.99 mV dec^−1^), and RuO_2_ (99.71 mV dec^−1^). It is noteworthy that, benefiting from the unique architecture, the Ni_1_/G‐FeO_x_ catalyst exhibits superior OER activity to most recently reported state‐of‐the‐art electrocatalysts (Figure 3e and Table S3). Electrochemical impedance spectroscopy (EIS) spectra showed that the Ni_1_/G‐FeO_x_ displayed the smallest semicircle. The results suggest that Ni_1_/G‐FeO_x_ exhibits better charge‐transfer kinetics with the electrolyte during the OER process (Figure S23). Based on the faster electron transfer rate of Ni_1_/G‐FeO_x_ relative to Ni_1_/FeO_x_, it can be inferred that the formed graphene facilitates rapid charge transfer during the OER [29, 30]. The electrochemical double‐layer capacitance (EDLC) of Ni_1_/G‐FeO_x_ was calculated as 4.7 mF cm^−2^, higher than Ni_1_/FeO_x_ and G‐FeO_x_ of 3.9 and 4 mF cm^−2^ (Figures 3c and S24). This indicates that the unique architecture endows the Ni_1_/G‐FeO_x_ with more available active sites [31]. Importantly, the Ni_1_/G‐FeO_x_ electrode exhibits excellent durability with no obvious current decay over 330 h at 1.5 V versus RHE (Figures 3d and S25), which is substantially superior to Ni_1_/FeO_x_, whose current density decayed by approximately 48% after only 22 h of continuous operation. The superior stability of Ni_1_/G‐FeO_x_ may arise from its distinctive heterostructure architecture, which provides effective protection against structural degradation and mitigates metal dissolution [32, 33, 34]. Besides, this self‐supporting electrode circumvents the issues associated with polymer binders in conventional slurry‐based electrodes, such as hindered mass transport and the blockage of exposed active sites.

To elucidate the mechanism behind the enhanced OER performance, XPS was conducted to compare the electronic structures of Ni_1_/G‐FeO_x_ and Ni_1_/FeO_x_ (Figure S26). Relative to Ni_1_/FeO_x_, Ni_1_/G‐FeO_x_ exhibits a positive shift in the Fe 2p peaks and an altered Ni^2+^/Ni^3+^ ratio, indicating graphene‐induced electronic modulation at both Fe and Ni sites. Furthermore, the O 1s spectrum of Ni_1_/FeO_x_ reveals a lower binding energy for lattice oxygen and a more pronounced hydroxyl‐related feature, suggesting enhanced surface hydroxyl adsorption or increased defect concentration upon graphene incorporation [35]. Collectively, these XPS results demonstrate that the graphene layer actively participates in interfacial electronic regulation, promoting charge redistribution and facilitating electron transfer between atomically dispersed Ni sites and the FeO_x_ substrate. Furthermore, the post‐OER XPS (Figure S27) show that the Fe^2+^ and Fe^3+^ species in Fe 2p binding energies and peak shapes remain nearly unchanged after durability testing, confirming the structural stability of the catalyst. A increase in the relative proportion of Ni^3+^ species is also observed, indicating surface activation that may contribute to the enhanced OER activity (Tables S4 and S5). Moreover, in situ Raman spectroscopy was performed to probe the structural evolution of Ni_1_/G‐FeO_x_ during the OER process (Figures S28 and S29). For Ni_1_/G‐FeO_x_, a pair of pronounced characteristic peaks emerged in the range of 400–600 cm^−1^ at 0.2 to 0.5 V (versus Ag/AgCl), which can be ascribed to the stretching vibrations of NiOOH [36, 37]. These results confirm the existence of partial high‐valence state Ni single atom centers in Ni_1_/G‐FeO_x_ during the OER process, consistent with XPS results. The existence of high valence state Ni centers is beneficial for the more favorable hydroxyl adsorption process and faster electron transfer [38].

**FIGURE 4:**
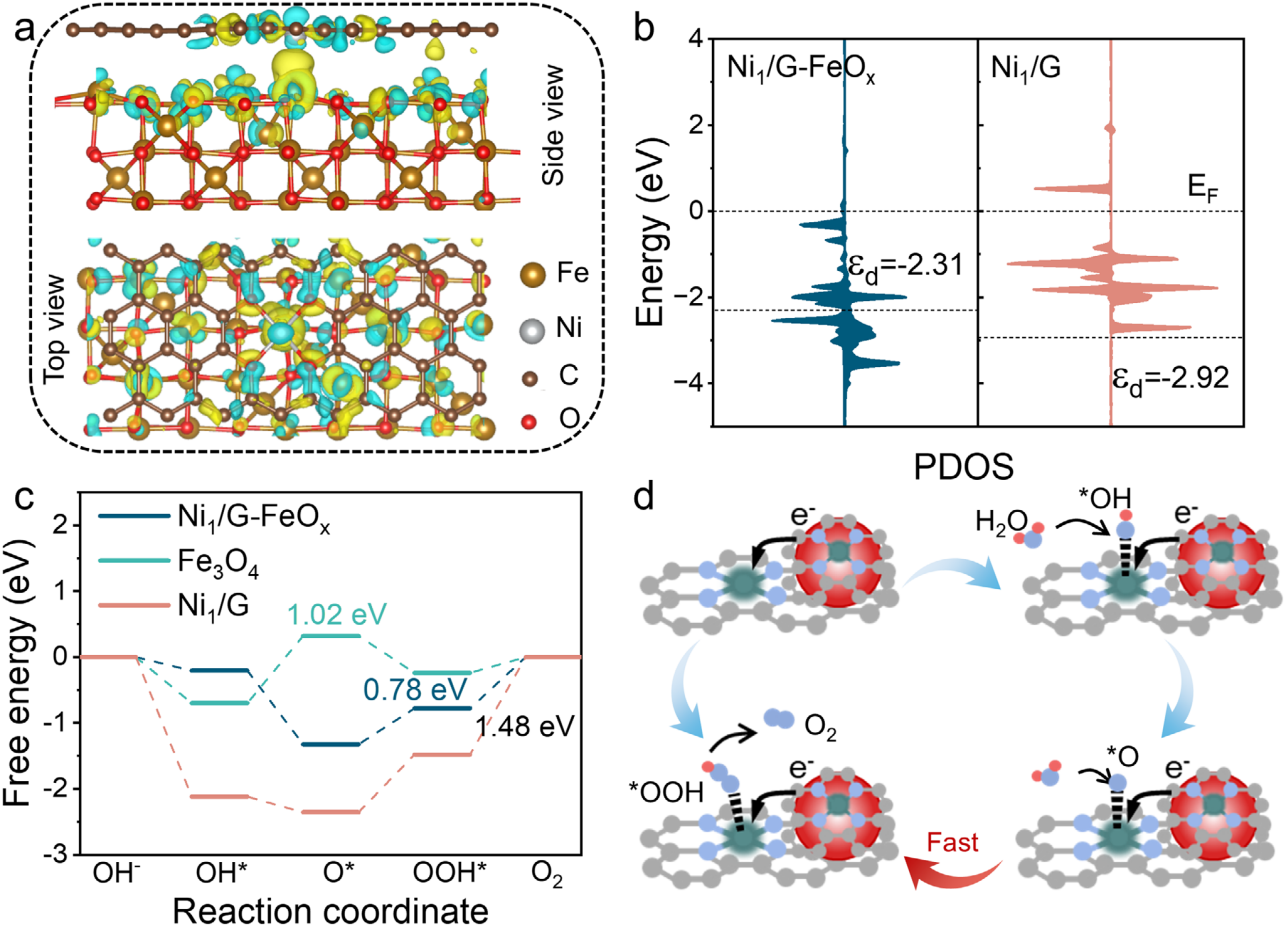
Theoretical insights into the OER reaction mechanism. (a) Charge density difference plot of Ni_1_/G‐FeO_x_. The yellow and blue areas correspond to the charge accumulation and the charge depletion. (b) Projected density of states of Ni atom in Ni_1_/G‐FeO_x_ and Ni_1_/G, respectively. (c) The free energy change of each elementary step on Ni_1_/G‐FeO_x_, Fe_3_O_4_ and Ni_1_/G surface. (d) Proposed OER mechanism over the optimized geometric structure of each intermediate on Ni_1_/G‐FeO_x_.

Density functional theory (DFT) calculations were further conducted to elucidate the OER mechanism of Ni_1_/G‐FeO_x_. First, the Ni_1_/G‐FeO_x_, Fe_3_O_4_, and Ni_1_/G models were constructed based on the experimental results. The charge density difference plot clearly reveals the interfacial electronic interactions in Ni_1_/G‐FeO_x_, as shown in Figure 4a. The top‐layer Ni_1_/G induces a strong spatial charge polarization across the interface. Significant charge redistribution is observed, where electron depletion occurs at the underlying Fe sites, while conspicuous electron accumulation (yellow regions) is localized around the bridging oxygen atoms at the interface. Below the graphene layer, the Fe_3_O_4_ surface shows a subtle charge rearrangement at the O sites, suggesting that the polarization generated at the Ni can be transmitted through graphene. This interlayer coupling gives rise to a polarized Ni_1_/G‐FeO_x_ interface that is conducive to catalytic activation. The density of states analysis indicates that the Ni atom in Ni_1_/G‐FeO_x_ possesses a higher d‐band center (−2.31 eV) in comparison with Ni_1_/G (−2.92 eV) (Figure 4b). The closer the d‐band center of the metal atom is to the Fermi level (*E*_F_), the more easily electrons will transfer from the d‐orbitals of the Ni atom to the adsorbed OOH*, which can strengthen the adsorption between Ni and OOH* [39].

Figure 4c compares the free‐energy profiles of the OER pathway over Ni_1_/G‐FeO_x_, Fe_3_O_4_, and Ni_1_/G. For pristine Fe_3_O_4_, the formation of the O* intermediate exhibits the largest free‐energy barrier (1.02 eV), indicating that it is the rate‐determining step. In contrast, Ni_1_/G‐FeO_x_ shows a significantly reduced energy barrier of only 0.78 eV for the final OOH* → O_2_ step, which is also the rate‐limiting step on this hybrid surface. Ni_1_/G exhibits the highest barrier (1.48 eV), indicating sluggish OER kinetics. These results clearly demonstrate that Ni_1_/G‐FeO_x_ possesses the smallest overall energy barrier among the three catalysts, leading to the most favorable OER thermodynamics and thus the lowest theoretical overpotential. The synergistic electronic coupling between the Ni single‐atom sites and Fe_3_O_4_ effectively optimizes the adsorption energetics of oxygen intermediates. Figure 4d further illustrates the four proton‐coupled electron‐transfer steps on the Ni_1_/G‐FeO_x_ surface, involving the sequential formation of OH*, O*, and OOH* species and the final release of O_2_.

**FIGURE 5:**
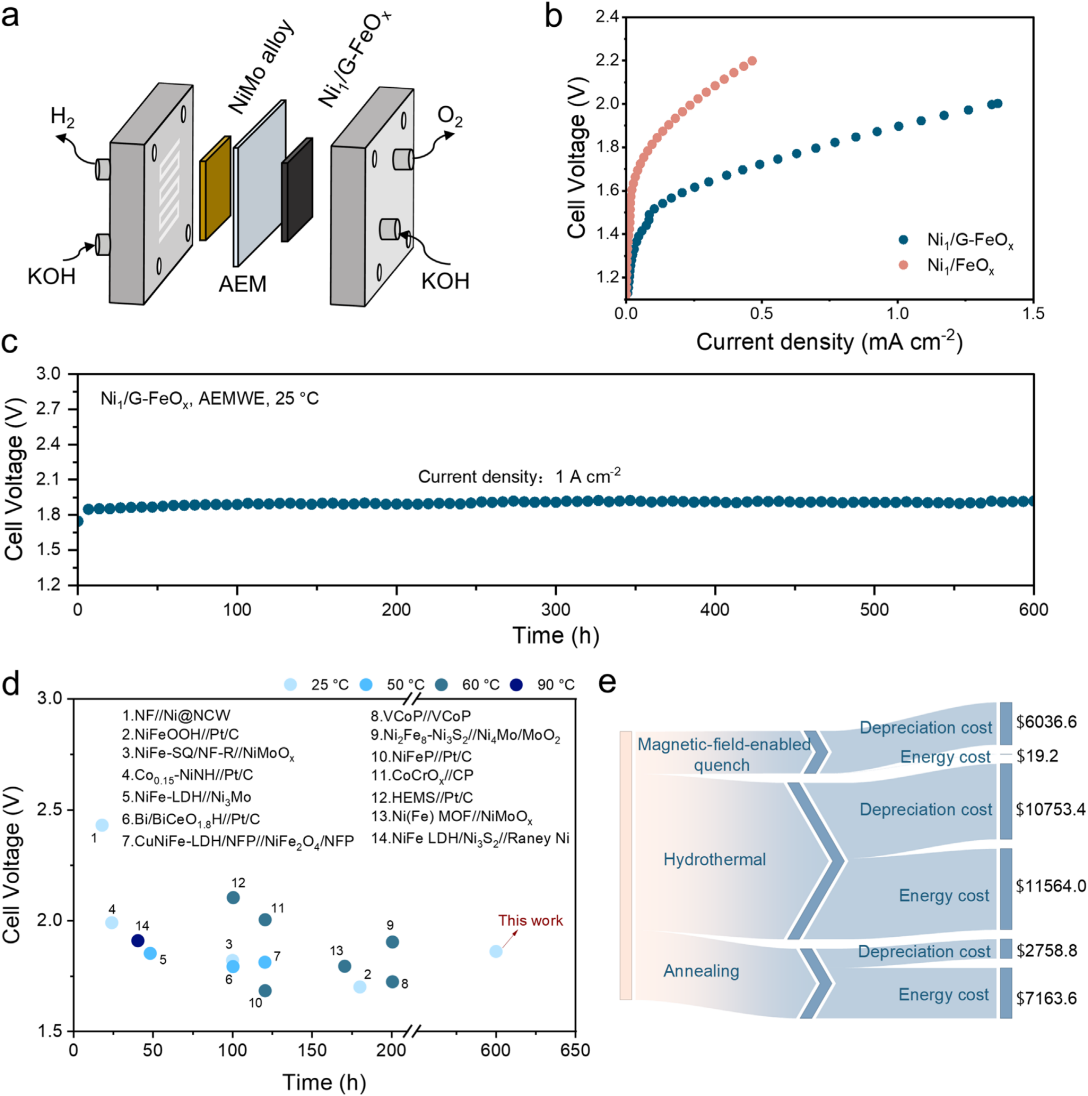
Electrochemical performance of the AEMWE device. (a) Schematic of the AEMWE device. (b) Polarization curves of the AEMWE cell using Ni_1_/G‐FeO_x_ as the anode (Cathode: NiMo alloy). (c) Long‐term durability of the AEMWE cell at 1 A cm^−2^. (d) Comparison of the stability time and cell voltage at 1 A cm^−2^ of Ni_1_/G‐FeO_x_ || NiMo alloy and other recently reported noble metal‐free AEMWE devices. The corresponding references are listed in Table S6. (e) Economic analysis of the depreciation cost and energy cost across different preparation methods.

Benefiting from its outstanding OER activity, the AEMWE device was assembled using Ni_1_/G‐FeO_x_ as the anode and NiMo alloy as the cathode (Figures 5a and S30). As shown in Figure 5b, the Ni_1_/G‐FeO_x_‐enabled AEMWE delivers remarkably low cell voltage of 1.86 V at 1000 mA cm^−2^ and can further reach ∼1.4 A cm^−2^ at 2 V, significantly outperforming the Ni_1_/FeO_x_ catalyst, which requires 2.0 V to reach only 250 mA cm^−2^. More importantly, the device maintains exceptional durability, operating steadily for over 600 h at an industrially relevant current density of 1000 mA cm^−2^ without noticeable performance degradation (Figure 5c). Notably, the Ni_1_/G‐FeO_x_ ‐based AEMWE surpasses most reported noble‐metal‐free systems (Figure 5d and Table S6), underscoring its strong promise for practical large‐scale hydrogen production.

To demonstrate the broad generality of the MFEQ strategy, we extended the synthesis to a range of transition and noble metal precursors, including Fe(NO_3_)_3_·9H_2_O, Co(NO_3_)_2_·6H_2_O, Ir(acac)_3_, Ru(acac)_3_, and Pt(acac)_2_. By simply replacing ethanol with other quenching solvents such as DMF, we successfully obtained a series of M_1_/G‐FeO_x_ (M═Fe, Co, Ir, Ru, Pt) SACs. The SEM, HAADF‐STEM images, together with the corresponding STEM‐EDS elemental mapping analyses (Figures S31–S37), collectively reveal the presence of isolated metal atomic sites, demonstrating that the target metal species are uniformly dispersed throughout the M_1_/G‐FeO_x_ framework in an atomically dispersed manner, without detectable metal clusters or nanoparticles. All these materials exhibit significantly enhanced OER performance compared with G‐FeO_x_ and Fe foam (Figures S38 and S39). Furthermore, replacing Fe foam with Ni foam enabled the formation of an Fe_1_/G‐NiO_x_ catalyst (Figure S40), which also delivers markedly superior OER activity (Figure S41). Collectively, these results highlight the excellent generality of the MFEQ method, demonstrating that rational selection of metal precursors, metal foam substrates, and organic solvents enables the rapid construction of a broad range of supported SACs.

To evaluate its practical applicability, a preliminary techno‐economic analysis (TEA) was carried out to assess the economic viability of the MFEQ process (Figure 5e). The TEA considered both depreciation and energy consumption costs. The results reveal that MFEQ offers a remarkable energy‐cost advantage, requiring only US$19.2 to synthesize 1 kg of catalysts, which is substantially lower than the costs associated with conventional hydrothermal (US$11 564.0) and high‐temperature annealing (US$7163.6) methods. Therefore, MFEQ represents a highly promising synthetic strategy with strong potential for large‐scale synthesis of highly efficient catalysts for hydrogen production.

## 3 Conclusion

Herein, we report an ultrafast, scalable, and low‐cost MFEQ method for synthesizing a broad range of SACs. As a proof‐of‐concept, the obtained Ni_1_/G‐FeO_x_ catalyst delivers outstanding OER performance, featuring a low overpotential of 200 mV at 10 mA cm^−2^ and excellent long‐term stability at 105 mA cm^−2^. Experimental and theoretical analyses reveal that the strong electronic coupling between the Ni_1_/G and FeO_x_ substrate effectively optimizes the adsorption energetics of oxygen intermediates, thereby accelerating OER kinetics. Furthermore, the Ni_1_/G‐FeO_x_‐enabled AEMWE device demonstrates a low operating voltage of 1.86 V and a remarkable durability of 600 h under industry‐level current densities. Techno‐economic analysis confirms that the MFEQ strategy offers substantial advantages in both energy consumption and overall cost for SAC fabrication. This work provides new insights into the efficient and low‐cost synthesis of versatile, supported SACs for electrochemical energy conversion applications.

## Author Contributions

**Shenghua Chen**: conceptualization, methodology, investigation, formal analysis, funding acquisition, writing – original draft, visualization, writing – review and editing, supervision. **Yaqiong Su**: investigation, validation. **Kaixin Liang**: investigation, data curation. **Yiqing Sun**: formal analysis, data curation. **Fangkun Fan**: validation. **Yifei Shen**: validation, data curation. **Lingyou Zeng**: formal analysis, validation. **Kai Xi**: formal analysis, data curation. **Chunhui Xiao**: visualization. **Xiaobo Zheng**: writing – original draft, writing – review and editing, methodology, data curation, investigation, funding acquisition, formal analysis, visualization. **Dingsheng Wang**: writing – review and editing. **Guoxiu Wang**: writing – review and editing, supervision. **Ya‐Ling He**: writing – review and editing, project administration.

## Conflicts of Interest

The authors declare no conflicts of interest.

## Supporting information




**Supporting File**: anie73373‐sup‐0001‐SuppMat.docx.

## Data Availability

The data that support the findings of this study are available in the Supporting Information of this article.
