# Supplementary material for: Magnetic‐Field‐Enabled Ultrafast Quench Synthesis of Single‐Atom Catalysts for Efficient Anion Exchange Membrane Water Electrolysis
Source: Angew Chem Int Ed Engl. 2026 Jul 8;65(37):e2977516. doi: 10.1002/anie.2977516 (PMC13548983; doi:10.1002/anie.2977516)
Supplement: Supplementary file 1 [file ANIE-65-e2977516-s001.docx]

**Supporting Information**

**Magnetic-Field-Enabled Ultrafast Quench Synthesis of Single-Atom Catalysts for Efficient Anion Exchange Membrane Water Electrolysis**

Shenghua Chen^[a]+*^, Yaqiong Su^[a]+^, Kaixin Liang^[b]+^, Yiqing Sun^[a]^, Fangkun Fan^[a]^, Yifei Shen^[a]^, Lingyou Zeng^[a]^, Kai Xi^[a]^, Chunhui Xiao^[a]^, Xiaobo Zheng^[c]*^, Dingsheng Wang^[d]^, Guoxiu Wang^[c]*^, Ya-Ling He^[a]*^

^[a]^ School of Chemistry, National Innovation Platform (Center) for Industry-Education Integration of Energy Storage Technology, School of Chemical Engineering and Technology, Xi'an Jiaotong University, Xi'an 710049, P. R. China. E-mail: shenghchen@xjtu.edu.cn; yalinghe@mail.xjtu.edu.cn

^[b]^ Shanxi Xinhua Chemical Equipment Research Institute Co., Ltd., Taiyuan 030003, P. R. China.

^[c]^ Centre for Clean Energy Technology, School of Mathematical and Physical Sciences, Faculty of Science, University of Technology Sydney, Sydney, New South Wales 2007, Australia

^[d]^ Department of Chemistry, Tsinghua University, Beijing 100084, P. R. China

^[+]^ These authors contributed equally to this work.

^*^Corresponding author: shenghchen@xjtu.edu.cn, XiaoboZheng.Zheng@uts.edu.au, Guoxiu.Wang@uts.edu.au, yalinghe@mail.xjtu.edu.cn

**Chemicals**

All chemicals were used as received without further purification. Nickel nitrate hexahydrate (Ni(NO_3_)_2_·6H_2_O, AR), iron(III) nitrate nonahydrate (Fe(NO_3_)_3_·9H_2_O, AR), cobalt nitrate hexahydrate (Co(NO_3_)_2_·6H_2_O, AR), ruthenium(III) acetylacetonate (Ru(acac)_3_, AR), platinum(II) acetylacetonate (Pt(acac)_2_, AR), and iridium(III) acetylacetonate (Ir(acac)_3_, AR) were purchased from Aladdin. Potassium hydroxide, ethanol, and DMF were obtained from Sinopharm Chemical Reagent Co., Ltd. Commercial RuO_2_ was supplied by Hesen Electric Co., Ltd. Fe foam and Ni foam were obtained from Qinghe Chengshuo Metal Materials Co., Ltd. Ultrapure water was used throughout all experiments.

**Materials synthesis**

**Synthesis of Ni_1_/G-FeO_x_.**

Ni_1_/G-FeO_x_ catalyst was prepared by the magnetic-field-enabled quench (MFEQ) synthesis method. In the first step, a piece of cleaned Fe foam (99.95 wt%, 2 cm x 1 cm) was annealed in air until its color turned red by a high-frequency induction heater (100~1100 kHz, 3KW, Zhongshan Better M&E Equipment Co., Ltd, China). The heating temperature is about 700 °C, which was monitored using a laser targeting infrared thermometer (Zhongshan Electronic Technology Co., Ltd; accuracy, ± 1 °C; response time, 5 ms). Then the annealed Fe foam was rapidly quenched in ethanol with Ni(NO_3_)_2_·6H_2_O at room temperature to grow Ni_1_/G-FeO_x_. The whole quenching process lasted a few seconds (Supplementary Movie). Finally, the resulting product was collected, washed and lyophilized.

**Synthesis of G-FeO_x_.**

For the synthesis of G-FeO_x_, pure ethanol without any metal salt was used as the liquid carbon source, while all other procedures were identical to those used for synthesizing Ni_1_/G-FeO_x_.

**Synthesis of Ni_1_/FeO_x_.**

For the synthesis of Ni_1_/FeO_x_, the liquid carbon source was replaced with an aqueous solution of nickel nitrate, and the remaining steps followed the same procedure as Ni_1_/G-FeO_x_.

**Synthesis of Fe_1_/G-FeO_x_.**

For the synthesis of Fe_1_/G-FeO_x_, Fe(NO_3_)_3_·9H_2_O was used as the metal precursor, with all other experimental steps unchanged from the Ni_1_/G-FeO_x_ synthesis.

**Synthesis of Ru_1_/G-FeO_x_.**

For the synthesis of Ru_1_/G-FeO_x_, the liquid carbon source was replaced with a DMF solution containing ruthenium (III) acetylacetonate, while the rest of the procedure remained identical to that of Ni_1_/G-FeO_x_.

**Synthesis of Ir_1_/G-FeO_x_ and Pt_1_/G-FeO_x_.**

Similarly, Ir_1_/G-FeO_x_ and Pt_1_/G-FeO_x_ were obtained by using DMF solutions containing iridium (III) acetylacetonate and platinum (II) acetylacetonate, respectively, with all other steps consistent with the Ni_1_/G-FeO_x_ synthesis protocol.

**Synthesis of Fe_1_/G-NiO_x_.**

For the synthesis of Fe_1_/G-NiO_x_, the Fe foam was changed into Ni foam; other procedures are the same as the synthesis of Fe_1_/G-FeO_x_.

**Characterization**

XRD was performed by using a Rigaku RU-200B X-ray diffractometer equipped with Cu K_α_ radiation (λ = 1.5406 Å). Raman spectra were recorded with a Raman spectrometer (JY HR800, 532 nm laser wavelength, 1 μm spot size). The laser power was below 2 mW to avoid laser-heating-enabled damage on the sample. SEM images were taken from Helios G4 CX (FEI, USA) at a working voltage of 2 kV. TEM was conducted on a Hitachi H-800 TEM. HRTEM images and EDS were recorded by a JEOL JEM-2100F with an acceleration voltage of 200 kV. HAADF-STEM images and corresponding elemental mapping were obtained using a JEOL JEM-200F transmission electron microscope operated at an accelerating voltage of 200 kV and equipped with a probe spherical aberration corrector. XPS spectra were collected on the SSI S-Probe XPS Spectrometer. For structural characterizations and property measurements, we transferred the catalyst sample from the Fe foam onto other substrates.

**Electrochemical measurements**

Experiments were conducted in a standard three-electrode system controlled by an electrochemical workstation (CHI 760e). The synthesized self-supporting electrode, an Ag/AgCl electrode, and Pt foam were used as the working, reference, and counter electrodes, respectively. The oxygen evolution reaction (OER) performance was evaluated by linear sweep voltammetry (LSV), which was obtained in an electrolyte of O_2_-saturated 1.0 M KOH with a scan rate of 5 mV/s. The Tafel slopes were calculated according to the Tafel equation η= b log(j/j_0_) based on the LSV curves, where η is the overpotential, b is the Tafel slope, j is the current density, and j_0_ is the exchange current density. The electrochemical double-layer capacitance (EDLC) was calculated from cyclic voltammetry (CV) curves recorded in a non-Faradaic potential region. By plotting the capacitive density at 1.28 V vs. RHE against the scan rate (20, 30, 40 and 50 mV/s), a linear trend was observed. The electrochemical impedance spectroscopy (EIS) was tested in open-circuit voltage for OER from 100 kHz to 0.1 Hz. The stability measurement for OER was tested by the chronoamperometry method at 1.5 V vs. RHE. All potential measurements were converted to the RHE according to the following formula:

*E*_RHE_ = *E*_Ag/AgCl_ + *E*^0^_Ag/AgCl_ + 0.059 pH.

**In-situ Raman spectra measurements.**

Raman spectra are obtained by Horiba LabRAM HR Evolution with a He/Ne laser of λ = 532 nm and 4.9 mW. The current and voltage signals are collected by an electrochemical workstation (Autolab PGSTAT 204) in a customized Teflon cell with 1.0 M KOH solution. The self-supporting monolithic electrode worked as the working electrode, an Ag/AgCl electrode as the reference electrode and a polished platinum wire as the counter electrode.

**Computational Details**

Density functional theory (DFT) calculations were carried out using the Vienna ab initio simulation package (VASP).^[1]^ The projector-augmented wave (PAW)^[2]^ approach was used to describe the ion-electron interactions with the frozen-core approximation. The energies and geometries of the most stable configurations were obtained through minimization of the total energy with respect to geometry by spin-polarized Perdew-Burke-Ernzerhof (PBE)^[3]^ exchange-correlation functional within generalized gradient approximation (GGA) calculations. The Kohn-Sham valence states were expanded in a plane-wave basis set with a cut-off energy of 400 eV. For the Brillouin zone integration, a Monkhorst-Pack 3×3×1 mesh was used. The dispersion correction method DFT-D3 with Becke-Jonson damping was used to describe the van der Waals (vdW) interaction. The DFT+U approach was employed, where U is a Hubbard-like term describing the on-site Coulombic interactions,^[4]^ in order to improve the description of localized states in Fe 3d. For Fe, a value of U=5.3 eV was adopted. Charge density difference (CDD, Δρ) plots were obtained by the following equation:

$$\Delta\rho=\rho_{AB}-\rho_{A}-\rho_{B}$$

where$\rho_{\mathrm{AB}}$represents the charge density of the whole system; while$\rho_{A}$and$\rho_{B}$ represents the charge density of the two subsystems.

**Techno-economic analysis**

To evaluate the economic potential of scaling up the synthesis of MFEQ, an economic analysis was conducted for this synthesis method, along with the conventionally used hydrothermal and calcination methods typically employed in synthesizing similar catalysts, and a comparison was made.^[5,6]^ The analysis focuses on the synthesis process, with particular emphasis on the heating steps which demand the highest energy consumption and place the greatest demands on equipment. The specific calculations are as follows:

**Equipment depreciation cost**

This cost represents the allocation of the equipment procurement price and maintenance cost over its total output throughout its lifecycle. The calculation method is as follows:

$$Equipment depreciation cost=\frac{\text{Equipment}\text{ }\text{procurement}\text{ }\text{price}\text{(\$)+}\text{Equipment}\text{ }\text{maintenance}\text{ }\text{price}\text{(\$)}}{\text{365*Lifetime(y)}\text{*}\text{Output Per Day(g)}}$$

The equipment procurement prices, maintenance costs, and service life data are sourced from manufacturers. The primary equipment for the hydrothermal method is an oven, with a lifespan of 15 years; the primary equipment for the calcination method is a tube furnace and an oven, both with lifespans set at 15 years; the service life of the high-frequency induction heating equipment used in the MFEQ method is set at 8 years. The daily output over the lifecycle is calculated based on the number of production cycles that can be completed per day, assuming the hydrothermal method can complete 0.5 cycles per day, and the calcination method can complete 2 cycles per day. Due to the extremely fast synthesis speed of the MFEQ method, the daily number of cycles is practically constrained by stirring efficiency and is set at 40 cycles per day. The output per cycle for each method is measured from experiments.

**Energy consumption cost**

This item calculates the total electricity cost of the power-consuming heating processes during synthesis. The calculation method is as follows:

$$Energy consumption cost=\frac{\text{Equipment Energy Consumption(kW)}\text{*}\text{Work Time}\text{(}\text{h)}\text{*}\text{Electric Price(\$/}\text{kWh}\text{) }}{\text{Output(g)}}$$

The energy consumption for stirring is uniformly calculated as 25 W for all three methods. The hydrothermal method uses an oven, involving a 6-hour heating phase, followed by a 12-hour constant-temperature drying phase, and finally a 2-hour gradual cooling phase. The average power during the heating and cooling phases is 1240 W, and the power during constant-temperature drying is calculated as 620 W. The calcination method uses a tube furnace for a 4-hour heating process, which includes 2 hours of heating and 2 hours of air cooling, with an average power consumption of 1250 W. An oven is then used for a 24-hour drying task. Since the tube furnace and the oven can operate simultaneously, the outputs of the two pieces of equipment are balanced to ensure identical daily output and uninterrupted operation, avoiding waste of production capacity. The single heating time for the MFEQ method is 5 seconds, with an average heating power of 730 W, and it allows for continuous heating without requiring a cooling process.

In addition, we have clarified all major assumptions and operational parameters used in the calculations. For example, Equipment lifetimes were defined based on manufacturer information:

Hydrothermal oven: 15 years

Tube furnace and drying oven for calcination: 15 years

High-frequency induction heating system for MFEQ: 8 years

Daily production cycles were estimated according to the practical synthesis throughput of each method:

Hydrothermal method: 0.5 cycles per day

Calcination method: 2 cycles per day

MFEQ method: 40 cycles per day

The heating power, operation time, and cooling conditions for each synthesis route were also explicitly described. For example:

Hydrothermal synthesis involves a 6 h heating stage, 12 h drying stage, and 2 h cooling stage.

The calcination route includes tube-furnace heating and long-time drying treatment.

The MFEQ process only requires ~5 s induction heating without additional cooling treatment.

Stirring power consumption and electricity cost assumptions were also added to ensure calculation consistency across all methods.

**Figures and captions.**

**Figure S1**. The synthesis schematic illustration of MFEQ.

A clean piece of iron foam was rapidly heated to 700 °C (~5 s) in air using a high-frequency induction heater and then immediately quenched in an ethanol solution containing Ni(NO_3_)_2_·6H₂O at room temperature, resulting in the rapid formation of Ni₁/G-FeO_x_.

**Figure S2**. (a, b) SEM images of the Fe foam at different magnifications.

**Figure S3**. (a, b) SEM images of G-FeO_x_ at different magnifications, (c) EDS spectrum, and (d) corresponding elemental mapping images of G-FeO_x_.

**Figure S4**. (a, b) SEM images of Ni_1_/G-FeO_x_ at different magnifications. (c) EDS spectrum and (d) corresponding elemental mapping images of Ni_1_/G-FeO_x_.

**Figure S5**. Schematic diagram of the synthesis of SACs via MFEQ.

**Figure S6**. (a, b) HAADF-STEM image of Ni_1_/G-FeO_x_ catalyst, (c) the corresponding fast Fourier transform (FFT) pattern and (d) measurement of interplanar spacing by averaging eight lattice fringes of the marked crystallite in (b).

**Figure S7**. (a, b) HAADF-STEM images of Ni_1_/G-FeO_x_ catalyst.

**Figure S8**. STEM-EDS elemental mapping images of Ni_1_/G-FeO_x_.

**Figure S9**. (a) The HAADF-STEM image of Ni_1_/G-FeO_x_. (b) EELS analysis of Ni_1_/G-FeO_x_. (c) The HAADF-STEM image of Ni_1_/G-FeO_x_. (d) The STEM-EDS line scan profile of the highlighted area in (c).

**Figure S10**. (a, b) HAADF-STEM images of the Ni₁/G-FeO_x_ catalyst. The inset shows the lattice-fringe line profiles used to determine the interplanar spacing by averaging five adjacent lattice fringes in the selected regions A and B.

**Figure S11**. XPS survey spectra of G-FeO_x_, Ni_1_/FeO_x_ and Ni_1_/G-FeO_x_.

**Figure S12**. WT plots for the Ni K-edge EXAFS signal for (a) Ni foil, (b) NiO, and (c) Ni_1_/G-FeO_x_.

**Figure S13**. The Ni K-edge FT-EXAFS fitting of Ni_1_/G-FeO_x_.

**Figure S14**. Fe K-edge XANES fitting curve of Ni_1_/G-FeO_x_.

**Figure S15**. Fe K-edge FT-EXAFS spectra of Ni_1_/G-FeO_x_, Fe_3_O_4_ and Fe foil.

**Figure S16**. The Fe K-edge FT-EXAFS fitting of (a) Fe_3_O_4_ and (b) Ni_1_/G-FeO_x_.

**Figure S17**. WT plots for the Fe K-edge EXAFS signal for (a) Fe foil, (b) Fe_3_O_4_, and (c) Ni_1_/G-FeO_x_.

**Figure S18**. Electrolytic cell device diagram.

**Figure S19**. LSV curves of Ni₁/G-FeO_x_ catalysts prepared with different quenching times (1, 2 and 3 times).

**Figure S20**. XRD patterns of Ni₁/G-FeO_x_ catalysts prepared with different quenching times (1, 2 and 3 times).

**Figure S21**. (a, b) SEM images of Ni₁/G-FeO_x_-2 at different magnifications, (c) EDS spectrum, and (d) corresponding elemental mapping images of the Ni₁/G-FeO_x_-2 catalyst prepared through two consecutive quenching processes.

**Figure S22**. LSV curves of Ni₁/G-FeO_x_ catalysts prepared with different Ni salt solutions.

**Figure S23**. Nyquist plots of Ni_1_/G-FeO_x_, Ni_1_/FeO_x_, G-FeO_x_ and Fe foam.

**Figure S24**. CV curves at different scan rates ranging from 10 mV s^-1^ to 50 mV s^-1^. (a) Ni_1_/G-FeO_x_, (b)Ni_1_/FeO_x_ and (c) G-FeO_x_. (d) The corresponding EDLC values.

**Figure S25**. SEM images of the Ni_1_/G-FeO_x_ after durability testing.

**Figure S26.** High-resolution Fe 2p, Ni 2p, and O 1s XPS spectra of Ni₁/FeO_x_ and Ni₁/G-FeO_x_: (a) Fe 2p, (b) Ni 2p, and (c) O 1s.

**Figure S27.** High-resolution (a) Ni 2p and (b) Fe 2p XPS spectra of Ni₁/G-FeO_x_ collected before and after durability testing.

**Figure S28**. Diagram of the in-situ Raman test setup.

**Figure S29**. In situ Raman spectra of (a) Ni₁/G-FeO_x_ and (b) G-FeO_x_ recorded during OER in 1.0 M KOH at applied potentials ranging from 0.2 to 0.5 V (vs Ag/AgCl).

**Figure S30**. Photographs of AEMWE.

**Figure S31**. (a, b) SEM images of Co_1_/G-FeO_x_ at different magnifications. (c) EDS spectrum and (d) the corresponding elemental mapping images of Co_1_/G-FeO_x_.

**Figure S32**. (a, b) SEM images of Pt_1_/G-FeO_x_ at different magnifications. (c) EDS spectrum of the Pt_1_/G-FeO_x_.

**Figure S33.** (a-b) HAADF-STEM images of Co_1_/G-FeO_x_. (c-d) STEM-EDS elemental mapping images of Co_1_/G-FeO_x_.

**Figure S34.** (a-b) HAADF-STEM images of Fe_1_/G-FeO_x_. (c-d) STEM-EDS elemental mapping images of Fe_1_/G-FeO_x_.

**Figure S35.** (a-b) HAADF-STEM images of Ir_1_/G-FeO_x_. (c-d) STEM-EDS elemental mapping images of Ir_1_/G-FeO_x_.

**Figure S36.** (a-b) HAADF-STEM images of Ru_1_/G-FeO_x_. (c-d) STEM-EDS elemental mapping images of Ru_1_/G-FeO_x_.

**Figure S37.** (a-b) HAADF-STEM images of Pt_1_/G-FeO_x_. (c-d) STEM-EDS elemental mapping images of Pt_1_/G-FeO_x_.

**Figure S38**. The LSV curves of the Fe foam, G-FeO_x_, Fe_1_/G-FeO_x_, Co_1_/G-FeO_x_ and Ni_1_/G-FeO_x_.

**Figure S39**. The LSV curves of Ru_1_/G-FeO_x_ and Ir_1_/G-FeO_x_.

**Figure S40**. (a, b) SEM images of Fe_1_/G-NiO_x_ at different magnifications. (c) EDS spectrum and (d) the corresponding elemental mapping images of Fe_1_/G-NiO_x_.

**Figure S41**. The LSV curves of Ni foam and Fe_1_/G-NiO_x_.

**Table S1**. Ni K-edge EXAFS fitting parameters for Fe₃O₄ and Ni₁/G-FeOₓ.

| Shell | *N^[a]^* | *R*(Å)*^[b]^* | *σ*^2^(Å^2^)*^[c]^* | Δ*E*_0_(eV)*^[d]^* | *R* factor |
| --- | --- | --- | --- | --- | --- |
| Ni-O | 3.74 ± 0.46 | 2.034 ± 0.012 | 0.006 ± 0.001 | -5.42 ± 1.5 | 0.0083 |

*^a^N*: coordination numbers; *^b^R*: bond distance; *^c^σ*^2^: Debye-Waller factors; *^d^*Δ*E*_0_: the inner potential correction. *R* factor: goodness of fit.

**Table S2**. Fe K-edge EXAFS fitting parameters for Fe₃O₄ and Ni₁/G-FeOₓ.

|  | Shell | *N^[a]^* | *R*(Å)*^[b]^* | *σ*^2^(Å^2^)*^[c]^* | Δ*E*_0_(eV)*^[d]^* | *R* factor |
| --- | --- | --- | --- | --- | --- | --- |
| Fe_3_O_4_ | Fe-O | 4 | 1.835 | 0.030 | -8.8 | 0.012 |
|  | Fe-O | 6 | 1.972 | 0.014 |  |  |
| Ni_1_/G-FeO_x_ | Fe-O | 1.3 | 2.038 | 0.01 | 4.59 | 0.013 |

*^a^N*: coordination numbers; *^b^R*: bond distance; *^c^σ*^2^: Debye-Waller factors; *^d^*Δ*E*_0_: the inner potential correction. *R* factor: goodness of fit.

**Table S3**. Summary of reported OER performances on different catalysts.

| **Electrocatalyst** | **Overpotentials (mV)**  **at 10 mA cm^-2^** | | **Tafel slope**  **(mV dec^-1^)** | **Reference** |
| --- | --- | --- | --- | --- |
| **Ni_1_/G-FeO_x_** | **200** | **63.39** | | **This work** |
| NiFe-LDH/Ni_3_S_2_/NM | 202 | 45.10 | | *Angew. Chem. Int. Ed.* **2025**, *64,* e202504972 |
| NiFeP | 210 | 54.50 | | *J. Am. Chem. Soc.* **2025**, *147,* 1334 |
| NiFeCoCrW_0.2_ | 220 | 36.80 | | *Nat. Commun.* **2025**, *16*, 3327 |
| MnZnFeCoNi | 221 | 48.78 | | *Sci. Adv.* **2025**, 10, 6758 |
| NiFe-ANR | 228 | 37.00 | | *Angew. Chem. Int. Ed.* **2023**, *62,* e202300478 |
| NiFe-PPy | 229 | 38.00 | | *Angew. Chem. Int. Ed.* **2024**, *63,* e202409628 |
| Mo-NiFeOOH | 242 | 23.00 | | *Nat. Commun.* **2022**, *13*, 2191 |
| Co-B@Co_3_O_4_/ Co_3_O_4_ NSs | 257 | 84.00 | | *J. Am. Chem. Soc.* **2024**, *146*, 7467 |
| 60Fe/NF | 257 | 57.00 | | *Adv. Energy. Mater.* **2023**, 1*3*, 2301921 |
| FeCoNiCuYP/C | 259 | 64.00 | | *Adv. Mater.* **2024**, *37*, 2410295 |
| Eu_2_O_3_/NiS_2_ | 260 | --- | | *J. Am. Chem. Soc.* **2024**, *146*, 5324 |
| Co_3_O_4_-VCO | 262 | 38.20 | | *J. Am. Chem. Soc.* **2023**, *145*, 2271 |
| Ni_2_P-CoCH/CFP | 270 | 36.00 | | *Angew. Chem. Int. Ed.* **2023**, *62,* e202302795 |
| Co-Fe-Ga-Ni-Zn | 370 | 71.00 | | *Nano Res.* **2022**, *15*, 4799 |
| CNF/FeNiCo | 445 | 71.50 | | *Adv. Mater.* **2023**, *35*, 202305222 |

**Table S4.** The ratio of Fe^2+^/Fe^3+^(n_Fe_) was obtained from XPS results.

|  | S_Fe(Ⅱ)_ | S_Fe(Ⅲ)_ | n_Fe(Ⅱ)/Fe(Ⅲ)_ |
| --- | --- | --- | --- |
| Ni_1_/G-FeO_x_ before OER | 36012.22 | 15706.65 | 2.29 |
| Ni_1_/G-FeO_x_ after OER | 35395.19 | 22549.64 | 1.56 |

**Table S5.** The ratio of Ni^2+^/Ni^3+^(n_Ni_) was obtained from XPS results.

|  | S_Ni(Ⅱ)_ | S_Ni(Ⅲ)_ | n_Ni(Ⅱ)/Ni(Ⅲ)_ |
| --- | --- | --- | --- |
| Ni_1_/G-FeO_x_ before OER | 9610.984 | 3022.975 | 3.17 |
| Ni_1_/G-FeO_x_ after OER | 14459.55 | 12785.67 | 1.13 |

**Table S6.** Comparison of the stability and cell voltage of recently reported noble metal-free AEMWE.

| **Anode** | **Cathode** | **T (^o^C)** | ***J* (mA cm^-2^)** | **Cell voltage (V)** | **Time (h)** | **Reference** |
| --- | --- | --- | --- | --- | --- | --- |
| **Ni_1_/G-FeO_x_** | **NiMo alloy** | **25** | **1000** | **1.86** | **600** | **This work** |
| VCoP | VCOP | 60 | 1000 | 1.72 | 200 | *Energy Environ. Sci.* **2022**, *15*, 1882 |
| NF | Ni@NCW | 25 | 1000 | 2.43 | 18 | *Adv. Mater.* **2024**, *36*, 2304917 |
| NiFe LDH/Ni_3_S_2_ | Raney Ni | 90 | 800 | 1.90 | 40 | *Angew. Chem. Int. Ed.* **2025**, *64*, e202504972 |
| NiFeP | Pt/C | 50 | 1000 | 1.68 | 120 | *J. Am. Chem. Soc.* **2025**, *147,* 1334 |
| Bi/BiCeO_1.8_H | Pt/C | 50 | 1000 | 1.79 | 100 | *Adv. Mater.* **2024**, *36*, 202314211 |
| CoCrO_x_ | CP | 60 | 500 | 2.00 | 120 | *Nat. Commun.* **2024**, *15*, 3416 |
| NiFe-LDH | Ni_3_Mo | 50 | 1000 | 1.85 | 48 | *ACS Catal.* **2023**, *13*, 11589 |
| HMS | Pt/C | 60 | 1000 | 2.10 | 100 | *Joul* **2024**, *8*, 2342 |
| NiFeOOH | Pt/C | 25 | 500 | 1.70 | 180 | *J. Am. Chem. Soc.* **2025**, *147,* 36763 |
| Ni(Fe) MOF | Pt/C | 60 | 1000 | 1.79 | 170 | *Angew. Chem. Int. Ed.* **2025**, *64*, e202413916 |
| NiFe-SQ/NF-R | Pt/C | 25 | 1000 | 1.82 | 100 | *Nat. Commun.* **2025**, *15*, 3470 |
| Co_0.15_-NiNH | Pt/C | 25 | 1000 | 1.99 | 24 | *Energy Environ. Sci.* **2024**, *17*, 1468 |

**Reference**

[1] G. Kresse, J. Hafner, “Ab initio molecular-dynamics simulation of the liquid-metal-amorphous-semiconductor transition in germanium” *Physical Review B* **49**, (1994): 14251.

[2] PE. Blöchl, “Projector augmented-wave method” *Physical Review B* **50**, (1994): 17953.

[3] JP. Perdew, K. Burke, M. Ernzerhof, “Generalized gradient approximation made simple” *Physical Review Letters* **77**, (1996): 3865.

[4] S. Dudarev, G. Botton, S. Savrasov, et al., “Electron-energy-loss spectra and the structural stability of nickel oxide: an LSDA+ U study” *Physical Review B* **57**, (1998): 1505.

[5] X. Wang, Y. Chen, F. Li, et al., “Site-selective protonation enables efficient carbon monoxide electroreduction to acetate” *Nature Communications* **15**, (2024): 616.

[6] X. Jiang, Z. Guo, J. Xu, et al., “Sulfur Bridge Geometry Boosts Selective Fe^Ⅳ^=O Generation for Efficient Fenton-Like Reactions” *Advanced Science* **12**, (2025): 2500313.
